# Trapeziectomy and Alternative Suspension Technique in Thumb Carpometacarpal Arthritis: Patient-Reported Outcome Measures

**DOI:** 10.1016/j.jhsg.2022.02.006

**Published:** 2022-03-26

**Authors:** Cecile M.C.A. van Laarhoven, Sophie Treu, Leonardo C.A. Claasen, Mark Van Heijl, J. Henk Coert, Arnold H. Schuurman

**Affiliations:** ∗ Department of Plastic, Reconstructive and Hand Surgery, University Medical Center Utrecht, Utrecht, the Netherlands; † Department of Surgery, Hand and Wrist Unit, Diakonessenhuis, Utrecht, the Netherlands; ‡ Department of Plastic, Reconstructive and Hand Surgery, Erasmus Medical Center, Rotterdam, the Netherlands; § Department of Trauma Surgery, University Medical Center Utrecht, Utrecht, the Netherlands

**Keywords:** Alternative suspension technique, Carpometacarpal joint, Osteoarthritis, PROMS, Trapeziectomy

## Abstract

**Purpose:**

For treatment of carpometacarpal thumb joint osteoarthritis, a trapeziectomy with an alternative suspension technique can be performed as the primary surgery or as the secondary after a failed primary surgery. This study evaluates the midterm follow-up (median, 54 months) for this technique using patient-reported outcome measures.

**Methods:**

After trapeziectomy, an alternative suspension technique is performed with a flexor carpi radialis tendon strip. Leaving the insertion intact, the strip is tunneled through a drill hole in the base of the first metacarpal and then through a drill hole in the second metacarpal neck and then sutured back onto itself. This suspends the first metacarpal to the shaft of the second metacarpal, creating a strong, V-shaped suspension. As the technique is performed in both the primary and secondary surgery, we analyzed both groups separately. As the primary outcome, we evaluated pain and function with the Patient-Rated Wrist and Hand Evaluation. Further, we evaluated the Disabilities of the Arm, Shoulder and Hand and Short Form 12 questionnaire scores from eligible patients. Finally, we correlated pain and function to quality of life.

**Results:**

The median Patient-Rated Wrist and Hand Evaluation score was 16.0 (interquartile range, 1.5–40.4) after the primary surgery and 46 (interquartile range, 34.0–75.5) after the secondary surgery. Patients after the primary surgery also scored better on the Disabilities of the Arm, Shoulder, and Hand questionnaire compared to patients after the secondary surgery. The Short Form 12 questionnaire physical scores were negatively correlated with the Disabilities of the Arm, Shoulder, and Hand questionnaire scores for the primary group (correlation coefficient, −0.468) and negatively correlated with the Patient-Rated Wrist and Hand Evaluation pain scores for the secondary group (correlation coefficient, −0.703).

**Conclusions:**

Trapeziectomy with this alternative suspension technique for treatment of carpometacarpal thumb joint osteoarthritis shows good patient-reported outcome measures for primary surgery and poor patient-reported outcome measures after the secondary surgery.

**Type of study/level of evidence:**

Therapeutic IV.

The main goals in the treatment of carpometacarpal thumb joint (CMC-1) osteoarthritis are pain reduction and long-lasting preservation of function. For advanced stages of osteoarthritis in CMC-1, many surgeons use trapeziectomy with ligament reconstruction and thumb suspension as the treatment of choice.^1^^,^^2^ Apart from trapeziectomy, many different ligament reconstruction and suspension or interposition techniques have been described in the literature.^3^, ^4^, ^5^, ^6^ These techniques are all designed to prevent proximal migration of the first metacarpal, with shortening of the thumb, and an adduction deformity of the thumb.^7^ Overall, these techniques show good results in terms of pain reduction after long-term follow-ups.^8^, ^9^, ^10^ Despite the suspension, the disadvantage of some of these techniques continues to be the proximal migration of the thumb metacarpal, with recurrence of the adduction position of the thumb.^7^ To provide a more dependable suspension with correction of the adduction posture of the thumb, an alternative suspension technique (AST) with a strip of flexor carpi radialis (FCR) tendon after trapeziectomy is described.^11^ After trapeziectomy, the FCR tendon strip is used to suspend the first metacarpal to the second metacarpal using a drill hole through the first metacarpal base, and a second drill hole more distally placed in the neck of the second metacarpal (Fig. 1). With this suspension technique, the first metacarpal is suspended on the shaft of the second metacarpal by placing the FCR tendon strip in a V-shaped suspension. This way, a stable and strong reconstruction of thumb height is created, which could prevent proximal migration in the gap after trapeziectomy. The thumb can be placed in an anatomically functional position with this suspension, and an overadducted position of the thumb can be corrected. This technique can be used in the primary cases for CMC-1 osteoarthritis as well as for a secondary surgery after the failure of a previous surgery for CMC-1 osteoarthritis. No results have been reported yet for this technique, and biomechanical studies that evaluate this technique are not available. To analyze the results after trapeziectomy with this AST for CMC-1 osteoarthritis, we conducted a cross-sectional study using patient-reported outcome measures (PROMs) to analyze the outcomes of pain function and quality of life after surgery. We assessed patients operated primarily as well as patients operated secondarily (after a previously failed surgery) with a trapeziectomy and AST, and our hypothesis is that this technique can be used for both indications with a satisfactory outcome. We assessed pain and function as the primary outcomes by analyzing PROMs.

**Figure 1:**
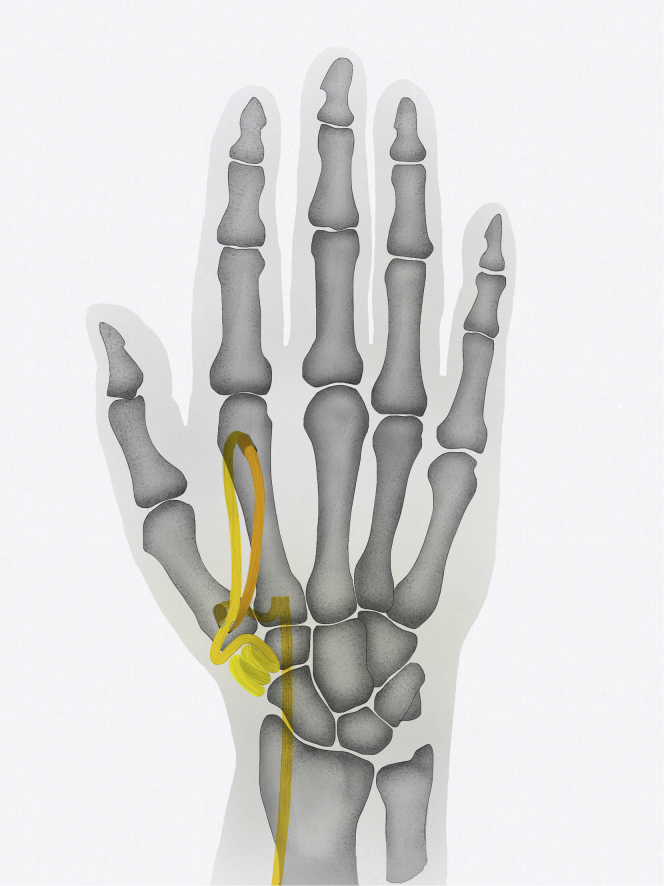
Surgical technique. After the trapeziectomy, a dorsovolar osseous tunnel is drilled at the base of the first metacarpal. Hereafter, a second tunnel is created in the dorsovolar direction in the second metacarpal neck, just proximal to the head of the second metacarpal. By this distal placement of the second tunnel, a V-shaped vector can be obtained with the suspension. A strip of the FCR tendon, with its insertion left intact, is used to suspend the first metacarpal to the second metacarpal in a V-shaped vector, because of the position of the second drillhole in the second metacarpal. Illustration by Marcus C.Y. Tong.

## Materials and Methods

### Study design

Between 2006 and 2015, 53 thumbs in 47 patients were treated with a trapeziectomy and AST for CMC-1 osteoarthritis. We used this cohort for a cross-sectional study design, because limited preoperative measurements were available. As the primary outcomes, we used the Dutch language version (DLV) of the Patient-Rated Wrist and Hand Evaluation (PRWHE) to assess pain and function.^12^^,^^13^ Secondarily, we assessed function with the DLV of the Disabilities of the Arm, Shoulder, and Hand questionnaire (DASH) and quality of life with the Short Form 12 questionnaire (SF-12).^14^^,^^15^

After getting approval from the institutional review board (Medical ethical committee University Medical Center Utrecht), we invited patients to participate in the study by mail or email. After obtaining written informed consent, we sent the patients 4 PROM questionnaires between December 2016 and March 2017. We reviewed all medical charts retrospectively for demographics, perioperative details, and complications.

### Patients

Trapeziectomy with AST was performed in the primary setting and after failed previous treatment for CMC-1 osteoarthritis with another surgical technique. Indications for surgery were painful CMC-1 osteoarthritis that did not respond to nonsurgical treatment (such as hand therapy and orthosis fabrication) and a radiographic stage 2–4 on the Eaton and Glickel radiographic classification scale for patients with no history of surgical treatment for CMC-1 osteoarthritis.^16^ For the patients who underwent previous surgical treatment, indications for this technique were renewed or persistent postoperative pain, caused by significant subsidence of CMC-1 with abutment on the scaphoid, that did not respond to rehabilitation therapy. Significant subsidence and abutment on the scaphoid, and whether this was provoking pain, were assessed by clinical examination and mostly confirmed with radiology. In this analysis, we included patients with CMC-1 osteoarthritis at Eaton and Glickel stage 2–4. We excluded the patients who were operated on with this technique for other indications (such as systemic degenerative arthritis, rheumatoid arthritis, or psoriatic arthritis), hyperlaxity syndromes, or Eaton and Glickel stage 1 at initial presentation.

This alternative procedure was specifically suitable for patients with evident subluxation or a tendency to subside. In the primary setting, the choice for this technique was made after shared decision making and after discussing all possible techniques. In case of a failed previous surgery, AST was the preferred treatment of choice.

This choice was based on subsidence after former surgery, with abutment of the first metacarpal on the distal scaphoid. A single surgeon performed all operations (A.H.S). We analyzed patients after the primary and secondary surgery separately. The patients with bilateral complaints had 2 operations, 1 for each hand. For the analysis, the questionnaires were filled twice, separately for the left and the right hand.

### Surgical technique

Spaans et al^11^ has previously described trapeziectomy with AST. Using a standard dorsal approach, a dorsal-radial incision at the base of the thumb helps expose the joint, avoiding injury to the superficial branch of the radial nerve and the radial artery. After a longitudinal capsulotomy, a trapeziectomy is performed. At the base of the first metacarpal, a dorsovolar osseous tunnel is drilled. Hereafter, another tunnel was created in the dorsovolar direction in the second metacarpal neck, just proximal to the head of the second metacarpal (Fig. 1). By this distal placement of the second tunnel, a V-shaped vector can be obtained with the suspension. The placement of the second tunnel was more distal than the placement described in the previous report by Spaans et al.^11^

On the volar side of the wrist, a 10–15-cm strip of one-third of the FCR tendon is harvested through 3 separate incisions, leaving the insertion intact at the volar base of the second metacarpal. In case of revision surgery, and if the FCR tendon strip was previously used in the primary surgery, a toe extensor is harvested and used for the suspension. The toe extensor is then secured on the insertion of the FCR tendon on the base of the second metacarpal to provide the same vector for the suspension as with the use of a FCR tendon strip. The tendon strip is passed through the tunnel in the first metacarpal (volar to dorsal), then over the adductor muscle and under the extensor pollicis longus (EPL) tendon, and subsequently through the distally placed tunnel in the second metacarpal (from dorsal to volar), after which the tendon is sutured back upon itself on the first metacarpal base. This construction lead to a strong, V-shaped suspension of the first metacarpal to the second metacarpal, and proximal migration should be prevented in case of an axial load on the first metacarpal. The thumb should be positioned in maximal radial and palmar abduction and an adducted position should be prevented. To prevent impingement of the first and second metacarpals, the remaining part of the FCR can be used as an interposition between the 2 metacarpal bases. The remaining end of the FCR tendon strip was placed in the space left by excising the trapezium. The capsule and skin were closed and a thumb spica cast was applied for 4 weeks. After 4 weeks, unloaded range-of-motion exercises were started, progressing to loaded range-of-motion exercises after 12 weeks.

### Measurements

We assessed pain and function, measured with PRWHE-DLV scores, as the primary outcomes.^12^ We used the DASH-DLV to compare our results with the literature.^14^ Because the DASH and PRWHE do not discriminate by side, we asked the patients who had bilateral surgery to fill in the questionnaire twice, separately for the left and right hand. Furthermore, quality of life was analyzed with the SF-12.^15^ The DASH-DLV, PRWHE-DLV, and SF-12 are 3 validated questionnaires. Furthermore, the patients were asked to choose from an 8-option list indicating why they requested the operation (Table 1).^17^

**Table 1:** Main Reason for Operation

Reason	Primary, n (%) n = 22	Secondary, n (%) n = 11
Reduce pain	18 (82)	10 (91)
Improve function	13 (59)	10 (91)
Improve activitites of daily living	8 (36)	7 (64)
Improve strength	6 (27)	5 (46)
Doing activities during leisure time	5 (23)	5 (46)
Able to work again	5 (23)	2 (18)
Improve appearance	2 (9)	2 (18)
Other	0 (0)	0 (0)

### Statistical analysis

We reported descriptive statistics as medians and interquartile ranges (IQRs) because of their nonnormal distribution, or as absolute values. The nonparametric Spearman’s rank correlation test was used to correlate the validated questionnaires with quality of life. When variables had a *P* value less than .05, they were considered to be statistically significant.

## Results

**Figure 2:**
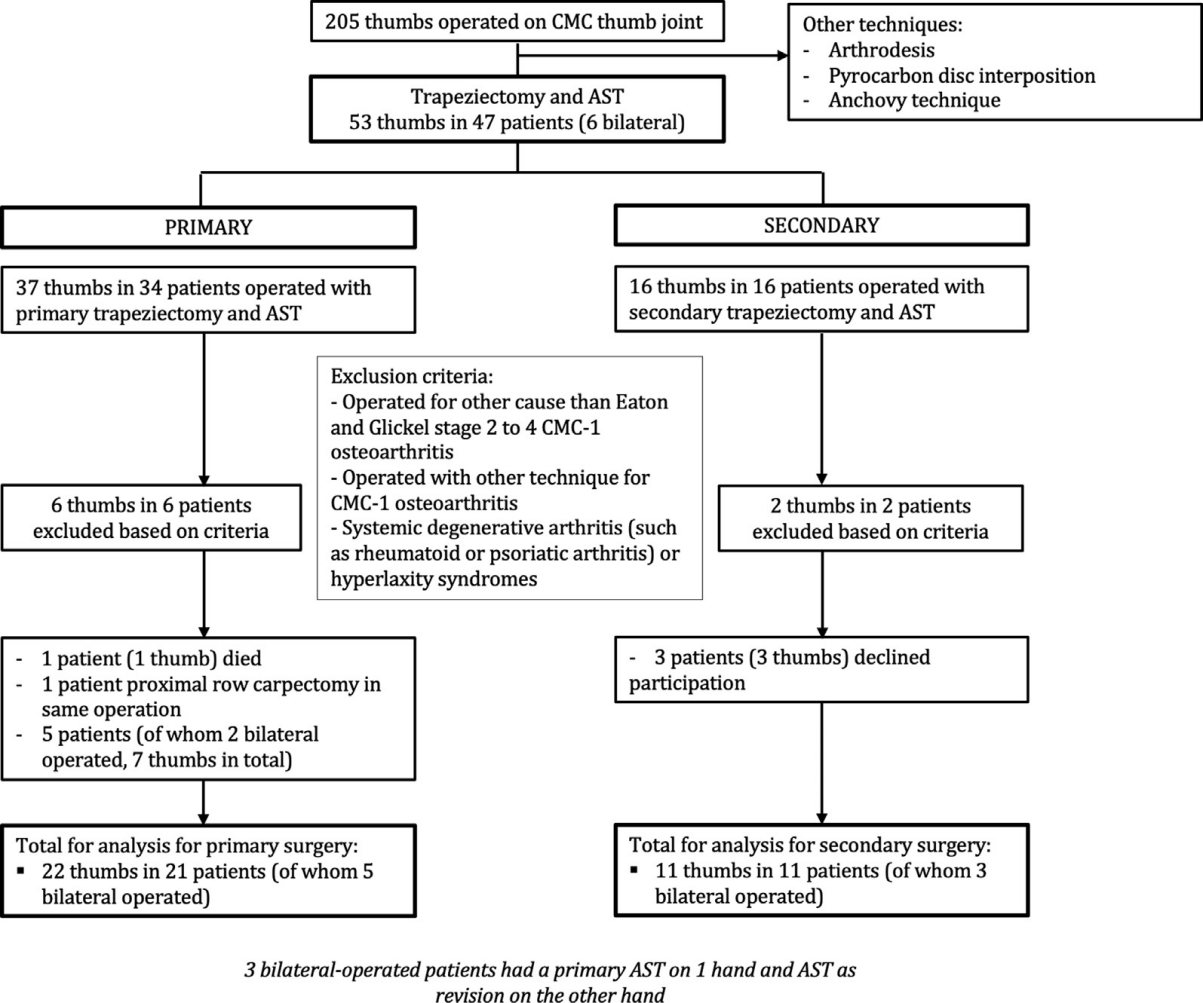
Flowchart of inclusion.

Of the 205 patients treated for CMC-1 osteoarthritis between 2006 and 2015, a total of 53 thumbs in 47 patients were treated with trapeziectomy and AST in our center (Fig. 2). Other techniques were arthrodesis, pyrocarbon disc interposition, and the anchovy technique, which were mainly performed for Eaton and Glickel stages 2 and 3. In 37 thumbs in 34 patients, trapeziectomy with AST was performed in a primary setting; in 16 thumbs in 16 patients, it was performed in a secondary setting. A total of 8 patients (6 in the primary group and 2 in the secondary group) met the exclusion criteria. One patient passed away before follow-up (primary) and 8 patients (10 thumbs) declined participation (7 thumbs in 5 patients in the primary group and 3 thumbs in 3 patients in the secondary group). In another patient, a proximal row carpectomy was performed during the same (primary) operation; therefore, this patient was excluded.

This resulted in a total of 29 patients (33 thumbs; 4 patients with bilateral operations in 2 surgeries at different times) for analysis (Fig. 2), and we evaluated their midterm follow-up results (median follow-up time, 54 months; range, 17–137 months).^18^ Table 2 shows their demographics and baseline characteristics. In 22 thumbs, the trapeziectomy with AST was performed as a primary operation for CMC-1 osteoarthritis; in 11 thumbs, it was performed as a secondary or salvage surgery, after a previous failed surgical CMC-1 treatment with subsidence of the first metacarpal.

**Table 2:** Baseline and Demographics

Demographics	No. of Patients	No. of Patients, Primary	No. of Patients, Secondary
Patients	29		
Thumbs	33	22	11
Single-side–operated patients	25	17	8
Bilateral-operated patients	4	5	3
Sex			
Male	6	5	1
Female	27	17	10
Age at treatment			
Years, median	59.6	58.9	62.6
IQR	50.3–66.4	50.8–66.1	49.9–70.7
Follow-up			
Months, median	54.0	53.5	61.0
IQR	32.0–78.5	26.5–71.3	33.0–100.0
Operated hand			
Left	16	11	5
Right	17	11	6
Operation dominant hand			
Yes	13	7	6
No	20	15	5
Eaton and Glickel stage			
Stage 2	1	1	NA
Stage 2–3	2	2	NA
Stage 3	2	2	NA
Stage 3–4	1	1	NA
Stage 4	16	16	NA
No stage because earlier (hemi)trapeziectomy or arthrodesis	11	0	11
First operation			
Anchovyplasty with hemitrapeziectomy	4	NA	4
Weilby (with trapeziectomy)	2	NA	2
Arthrodesis	1	NA	1
Trapeziectomy with tendon interposition	2	NA	2
Pyrocarbondisc interposition	1	NA	1
Hemitrapeziectomy	1	NA	1

NA, not applicable.

Table 3 presents the outcomes of the PRWHE, DASH, and SF-12. For all PROMs, the primary group showed better scores than those shown by the secondary group. For our primary outcome, the median PRWHE score after the primary surgery was 16.0 (IQR, 1.5–40.4) and, the median score after the secondary surgery was 46.0 (IQR, 34.0–75.5).

**Table 3:** Results of PROM Questionnaires for Primary and Secondary Surgery∗

PROMs	Primary Surgery			Secondary Surgery		
	Median	IQR	n	Median	IQR	n
PRWHE†						
Total	16.0	1.5–40.4	22	46.0	34.0–75.5	11
Function	4.3	0.0–21.3	22	21.0	15.0–37.5	11
Pain	3.0	0.0–24.0	22	26.0	9.0–41.0	11
DASH‡						
Preoperative	55.0	29.2–61.7	11			
Final follow-up	11.7	0.62–35.4	22	45.8	31.7 - 65.2	11
SF-12§						
Mental	53.8	34.0–58.1	22	49.8	35.3–59.6	9
Physical	37.7	32.3–51.4	22	32.5	26.1–36.2	9

∗ Data are given in medians, because of nonnormal distribution of data.

† Scores on the PRWHE range from 0 to 50 for pain and function, with 0 indicating the best outcome and 50 indicating the worst outcome. Total scores range from 0 to 100, with 0 indicating the best outcome and 100 indicating the worst outcome.

‡ Scores on the DASH range from 0 to 100, with 0 indicating the best outcome and 100 indicating the worst outcome.

§ The SF-12 for mental and physical health was developed for a mean of approximately 50 with an SD of 10.

Table 1 presents the main reasons why patients chose to have the operation, derived from the 8-option list. Patients scored pain as the main reason for operation in both the groups (82% for the primary and 91% for the secondary group). For the secondary group, the patients also scored function as an important factor for reoperation, which showed less importance in the primary group (secondary group, 91%; primary group, 59%).

There was a significant, negative correlation for the SF-12 physical score and the DASH questionnaire for the primary group (Table 4). For the secondary group, there was a significant, negative correlation for SF-12 physical score and the PRWHE pain score. We were not able to demonstrate any other significant correlations

**Table 4:** Correlations Between DASH, PRWHE, and SF-12 Scores (Spearman’s rank correlation)

SF-12 questionnaire	Group	PRWHE Total	PRWHE Pain	PRWHE Function	DASH Total
SF-12 mental	Primary	CC, −0.224 *P* = .32	CC, −0.162 *P* = .47	CC, −0.278 *P* = .21	CC, −0.396 *P* = .07
	Secondary	CC, 0.067 *P* = .87	CC, 0.318 *P* = .40	CC, −0.050 *P* = .90	CC, −0.201 *P* = .60
SF-12 physical	Primary	CC, −0.255 *P* = .25	CC, −0.311 *P* = .16	CC, −0.274 *P* = .22	CC, −0.468∗ *P* = .03
	Secondary	CC, −0.633 *P* = .07	CC, −0.703∗ *P* = .04	CC, −0.293 *P* = .44	CC, −0.485 *P* = .19

CC, correlation coefficient.

∗ Correlation is significant at the .05 level (2-tailed).

Two patients experienced postoperative complications. The first patient developed persistent pain after surgery and underwent a resection of the distal pole of the scaphoid after trapeziectomy and AST. A second patient developed a complex regional pain syndrome.

During the operation for the AST, another intervention was performed in 1 patient. This patient underwent an arthrodesis of the first metacarpophalangeal joint in the same operation.

## Discussion

At the midterm follow-up, our study shows low pain scores and good function, as measured with the PRWHE, together with excellent satisfaction after trapeziectomy with AST for CMC-1 osteoarthritis in the primary surgery. After the secondary or salvage surgery, the PRWHE and DASH outcomes were poor.

The aim of this study was to subjectively assess the results of a trapeziectomy with an AST. This specific technique was designed to improve thumb height with a suspension that could reduce the chance to subsidence, with the intention of alleviating pain, restoring joint stability, and increasing function of the thumb. We measured function and pain with the validated PRWHE and DASH questionnaires.

Other studies, which reported on PROMs after different techniques of trapeziectomy followed by a ligament reconstruction or suspension, reported higher scores of the DASH and PRWHE than those reported for the primary group after AST, suggesting better function and less pain after AST in this study at the midterm follow-up.^8^^,^^19^ In addition, for trapeziectomy, the reported DASH scores in literature for medium- to long-term follow-up are higher than those for the primary group after AST.^19^^,^^20^ Furthermore, De Smet et al^21^ found a significant correlation between the DASH scores and the functional and subjective outcomes for trapeziectomy with and without a ligament reconstruction and tendon interposition (LRTI), which is in line with our results.

A randomized controlled trial by Marks et al^22^ compared trapeziectomy with LRTI using an FCR tendon strip for suspension-interposition arthroplasty to an interposition allograft. At the 1-year follow-up, the DASH and SF-12 scores were presented. Their DASH scores were less positive than those for our primary group, whereas their SF-12 scores were better than those seen in our study population. Because there were no preoperative SF-12 responses available in our study, our results are difficult to interpret. Additionally, our results were taken at different points of follow-up, which makes comparison to other results more difficult.

When comparing the outcomes of the primary and secondary surgery groups, the primary group scored much better on the PROMs. This trend of mediocre-to-poor outcomes on PROMs after the secondary CMC-1 surgery for osteoarthritis has been described before.^23^, ^24^, ^25^, ^26^ The reason patients sought secondary treatment was mainly pain. The median pain score of the PRWHE was 26 (IQR, 9.0–41.0) for the secondary group after surgery, in contrast with the primary group’s median score of 3 (IQR, 0–24). This was also seen for the DASH score, for which the secondary group scored poor results when compared with other primary and secondary surgery results.^21^, ^22^, ^23^, ^24^ Unfortunately, no baseline results were available in our study group; hence, we could not draw any conclusions from these outcomes. These poor outcomes after the secondary surgery suggest that expectations should be adjusted and discussed with the patient. Furthermore, these results on the PROMs question the indication of this technique for secondary surgery for carpometacarpal osteoarthritis.

Interestingly, for the primary group, pain was the main reason for operation, whereas for the secondary group, pain and function were the main reasons for operation. Despite function and pain being scored as equally important to those undergoing the secondary operation, the results on function and pain, as measured by the PRWHE, were poor after the secondary surgery, which suggests neither better function nor less pain after the surgery. However, without preoperative scores, it is difficult to know how much these patients improved.

The DASH scores and quality of life, as measured with the SF-12 physical score, showed a negative correlation: that is, a lower DASH score (better function) correlated with a higher SF-12 physical score (higher physical quality of life). The secondary group showed a negative correlation between the PRWHE pain score and the SF-12 physical score: that is, better physical quality of life when less pain was noted. This suggests that the physical quality of life is more correlated to pain for patients after the secondary surgery than for patients after the primary surgery. This correlates with the outcome that a higher percentage of people in the secondary surgery group choose to have a secondary surgery because of pain (Table 1).

The study has several limitations. Unfortunately, in our study the preoperative subjective measures were insufficient and therefore could not be included. Hence, we could not determine how much the patients improved from their preoperative status. Furthermore, we did not perform measurements of thumb subsidence; hence, we could not correlate the PROMs to migration of the thumb, range of motion, or strength. Another limitation was that no postoperative radiographs were obtained, and a radiographic analysis of the suspension could not be performed.

In conclusion, this study shows similar results when compared with other studies. Future studies should focus on radiology in combination with clinical outcomes to demonstrate the benefits of this technique.

Good outcomes were found after the primary AST and poor outcomes were found for the secondary AST on midterm PROMs with the PRWHE, DASH, and SF-12. This suggests that AST is suitable for the primary surgery and less suitable for the secondary surgery. Patient expectations should be adjusted accordingly.
